# Why do Argos satellite tags stop relaying data?

**DOI:** 10.1002/ece3.7558

**Published:** 2021-05-01

**Authors:** Graeme C. Hays, Jacques‐Olivier Laloë, Alex Rattray, Nicole Esteban

**Affiliations:** ^1^ School of Life and Environmental Sciences Deakin University Geelong Vic. Australia; ^2^ Faculty of Science and Engineering Swansea University Swansea UK

**Keywords:** animal movement, Argos, Fastloc‐GPS, home range, migration, mortality, satellite tracking, telemetry

## Abstract

1. Satellite tracking of animals is very widespread across a range of marine, freshwater, and terrestrial taxa. Despite the high cost of tags and the advantages of long deployments, the reasons why tracking data from tags stop being received are rarely considered, but possibilities include shedding of the tag, damage to the tag (e.g., the aerial), biofouling, battery exhaustion, or animal mortality.
2. We show how information relayed via satellite tags can be used to assess why tracking data stop being received. As a case study to illustrate general approaches that are broadly applicable across taxa, we examined data from Fastloc‐GPS Argos tags deployed between 2012 and 2019 on 78 sea turtles of two species, the green turtle (*Chelonia mydas*) and the hawksbill turtle (*Eretmochelys imbricata*).
3. Tags transmitted for a mean of 267 days (*SD* = 113 days, range: 26–687 days, median = 251 days). In 68 of 78 (87%) cases, battery failure was implicated as the reason why tracking data stopped being received. Some biofouling of the saltwater switches, which synchronize transmissions with surfacing, was evident in a few tags but never appeared to be the reason that data reception ceased.
4. Objectively assessing why tags fail will direct improvements to tag design, setup, and deployment regardless of the study taxa. Assessing why satellite tags stop transmitting will also inform on the fate of tagged animals, for example, whether they are alive or dead at the end of the study, which may allow improved estimates of survival rates.

## Graphical abstract

We show how information relayed via satellite tags can be used to assess why tracking data stop being received. Assessing why satellite tags stop transmitting will inform on the fate of tagged animals, for example, whether they are alive or dead at the end of the study, thereby allowing improved estimates of survival rates and areas where animals may be experiencing high mortality. Furthermore, objectively assessing why tags fail will direct improvements to tag design, setup, and deployment regardless of the study taxa.

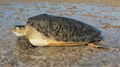

## 1 INTRODUCTION

Animal tracking studies continue to proliferate across taxa and habitats, with satellite tracking using the Argos system being very widely used (Hussey et al., 2015), with several 1000s of tags deployed each year (e.g., Hays & Hawkes, 2018). Satellite tracking is particularly useful for examining the long‐term movements of animals that range widely, including many birds, pelagic fish, sea turtles, and marine mammals (Hussey et al., 2015). The technique has led to a number of seminal advances including how animals use large areas such as ocean basins (Bailey et al., 2012), how they navigate during long migrations (Hays, Cerritelli et al., 2020), and the threats they face such as interactions with global fishing fleets (Fossette et al., 2014). As the cost of individual tags is relatively high (typically a few thousand US dollars), it is important to maximize data acquisition, for example, through long‐term deployments (Williams et al., 2020). Indeed, attempts to estimate the space of use of animals from tracking data are often constrained by limited tracking durations, with studies often suffering from “tagging site bias,” which is the term used to describe how sites closer to tagging sites tend to artificially emerge as high‐use areas (Hays, Rattray et al., 2020). Maximizing the duration of tracking is typically advantageous because it provides a truer picture of space use distant from the tagging sites (O'Toole et al., 2020). Yet despite the cost of tags and the advantages of long‐term tracking, it is surprising that the reasons why tags stop relaying data are rarely considered explicitly.

Over 10 years ago, it was shown how data relayed via the tags themselves might be used to assess the reasons for tag failure (Hays et al., 2007). Of the reasons why tags may stop relaying tracking data, animal mortality is perhaps the only one that has received much attention, with a number of studies across taxa including turtles, birds, fish, and mammals inferring mortality rates from satellite tagging data (Byrne et al., 2017; Hays et al., 2004; Henderson et al., 2020; Klaassen et al., 2014). However, there are a number of other reasons why data relay from satellite tags may stop, including the tag detaching, damage to the tag (the Argos antenna is often particularly vulnerable), biofouling, and battery exhaustion (Hays et al., 2007). Objective analysis of the likely reasons for tag failure may help refine tag design and setup. For example, put simply if biofouling of tags is a key issue, then efforts can be directed to reduce the rate of biofouling, for example, by using antifouling paint. Alternatively, if battery failure is an issue, efforts can focus on improved battery management. Here, we use a comprehensive dataset from state‐of‐the‐art Fastloc‐GPS Argos satellite tags to objectively examine the reasons why data relay from tags ceases. In this way, we outline general approaches that may help direct future improvements to satellite tracking studies across a broad range of taxa. The approaches we detail here may be used generally with Argos tags that are widely deployed across many taxa including marine mammals, sea turtles, and seabirds (Hussey et al., 2015).

## 2 MATERIALS AND METHODS

### 2.1 Satellite tag attachment

**FIGURE 1:**
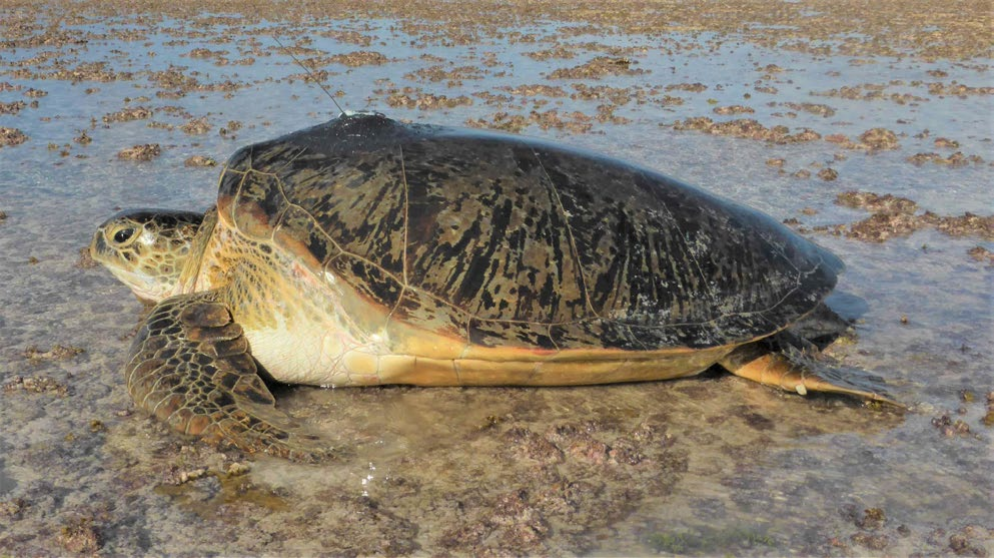
An adult green turtle (*Chelonia mydas*) equipped with a satellite tag. Sea turtles, in common with a broad range of taxa including mammals, birds, and fish, have been the subject of satellite tracking studies with many 1000s of satellite tags deployed around the world. Here, we objectively assess reasons why tags stop relaying data, developing approaches that can be used widely across tracking studies with different taxa. Photo credit: Nicole Esteban/Graeme Hays

We attached Wildlife Computers Fastloc‐GPS Argos tags (SPLASH10 units; Wildlife Computers, Seattle, Washington) to adult and immature green turtles (*Chelonia mydas*) and hawksbill turtles (*Eretmochelys imbricata*) in the Chagos Archipelago (7.43°S, 72.46°E) (Figure 1), where turtles are fully protected (Hays et al., 2020). Two models of tags were used: a larger unit (SPLASH10‐BF‐296C) with four AA lithium thionyl chloride batteries and a smaller unit (SPLASH10‐BF‐297B) with two AA lithium thionyl chloride batteries. All the tags (i.e., both large and small models) were programmed in the same way, and all tags included a GPS receiver. The only difference between the two tag models was the number of batteries and hence physical size of the tag. Tags were programmed to make a maximum of 250 Argos transmissions per day and to attempt acquisition of GPS ephemeris (a GPS “snapshot”) every 15 min, that is, 96 times per day. Note both models were conventional Argos tags but with the addition of a GPS receiver, so that data are relayed via Argos that allows Fastloc‐GPS locations to be obtained, in addition to Argos locations. So, the approaches we detail below can be applied to any Argos tags.

Immature turtles were captured in the lagoon on Diego Garcia, while adult female turtles were located on nesting beaches. Tag attachment is described in detail in Esteban et al. (2017) and Hays and Hawkes (2018), including a video of attachment (https://www.frontiersin.org/articles/10.3389/fmars.2018.00432/full#supplementary‐material). Prior to deployment, the sides and top surfaces of each tag were lightly sanded and then painted with two coats of antifouling paint (Trilux 33, International). After restraining a turtle, the carapace was cleaned to remove epibionts and grease. The tag was then embedded in quick setting epoxy (Pure‐2K; Powers Fastening Innovations or Pure 150‐PRO, DEWALT, which are the same products but with different branding). When the epoxy had almost cured, it was painted with antifouling paint along with any exposed part of the tag receiving a third coat.

### 2.2 Relay of Argos data via a ground station

In addition to relay of data via Argos satellites, data were also relayed via a ground‐receiving station called a Mote (Wildlife Computers, Seattle, Washington) that we installed on Diego Garcia, the largest island in the Chagos Archipelago and where all but one of the tags were deployed. The Mote is a fully autonomous ground‐based receiving station. In our configuration, the Mote consisted of two directional receiving antennae tuned to receive Argos message (401 MHz), a solar panel with battery for a constant supply source, and a signal processor for archiving the received Argos messages. The Mote antennae were positioned on the top of a 40 m tower to maximize the line‐of‐sight receiving distance, with the solar panel and signal processor at ground level for ease of servicing and data download. Data from the Mote were downloaded via a USB connector and processed through the Wildlife Computers data portal (http://my.wildlifecomputers.com/). The value of the Mote was that for turtles that remained in line of sight, more data were received than from the Argos satellites alone. Most of the immature turtles, all of which were equipped with the smaller model of tag, stayed at Diego Garcia and remained within line of sight of the Mote. All the nesting turtles, which were generally equipped with the larger model of tag, migrated away from Diego Garcia and so then data were only received via the Argos satellites.

### 2.3 Assessing end of battery life

Tags were programmed to primarily relay Fastloc‐GPS information, so that the best reconstruction of tracks would be possible. However, tags additionally relayed some diagnostic information. We assessed whether batteries had become exhausted in two ways. (a) First, battery voltage of the tags was relayed periodically. Information from the tag manufacturer was that a battery voltage of >3.0 V indicated that the batteries were still working well, but when the voltage dropped steeply to <3.0 V this indicated battery exhaustion. We therefore inferred that batteries were exhausted when battery voltage dropped below 3.0 V or when battery voltage dropped consecutively in 2 or more of the final tag status transmissions. (b) Second, tags periodically relayed a count of the number of Argos transmissions they had made. Lithium thionyl chloride batteries tend to lose voltage very quickly and so in many cases we expected not to receive data concerning the drop in voltage before tags stopped transmitting. Therefore, we assessed the total number of Argos transmissions made for those tags where the drop in battery voltage was relayed. Then, we used this total number of transmissions as an indicator of whether the batteries were similarly exhausted in the remaining tags.

### 2.4 Failure of the saltwater switches (biofouling)

The saltwater switches are three stainless steel contacts on the surface of the tag. When the tag functions correctly and is submerged in water, an electrical current connects at least two out of the three saltwater contacts and as a result the tag becomes dormant. When the turtle surfaces and the tag emerge from the water, the saltwater switches are exposed to air and the electrical circuit is broken. Then, immediately the tag attempts to acquire a GPS ephemeris or makes an Argos transmission. Two values with arbitrary units describe the states of the saltwater switches, the maximum dry state, and the minimum dry state (i.e., the wet state). These saltwater switch states are relayed via Argos. When the switches were operating perfectly, the maximum dry state value was around 200–250 and the wet state value around 50. In the event that the saltwater switches become biofouled, they tend to remain wet when the turtle surfaces and so the maximum dry state value drops. If the “dry” state drops from 200 and converges to the same value as the wet state (around 50), then the tag is no longer able to perceive when it is at the surface and Argos transmissions will cease. Once a tag stops relaying data, status information about the saltwater switches is no longer received. So, an indication that failure of the saltwater switch caused the cessation of data relay would be a preceding progressive decline in the dry state value so that it has converged very close to the wet state value. However, if there was still a clear difference between “dry” and “wet” states when the last data were received, then other reasons for the cessation of data relay are implicated.

## 3 RESULTS

### 3.1 Duration of tracking

**FIGURE 2:**
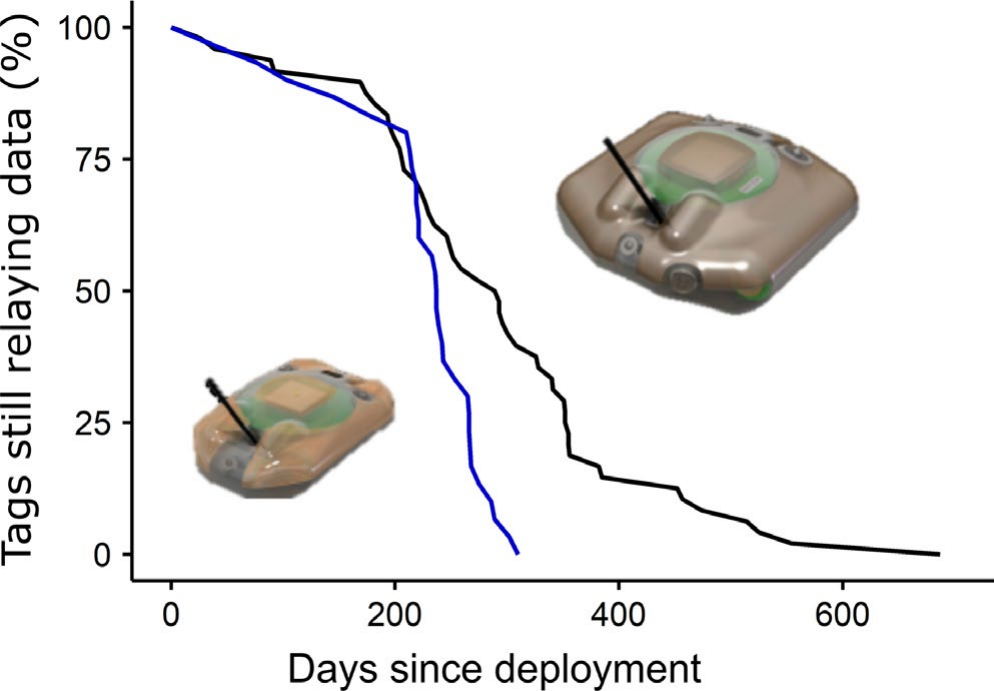
For the larger (black line) and smaller (blue line) models of tag, the proportion of tags still transmitting at different times after deployment. For example, on the day of deployment 100% of tags of both types were working, then 200 days after deployment about 80% of tags were still relaying data after which time this percentage started to decline steeply. The median durations of tracking for the larger and smaller models were 291 days (*n* = 48) and 237 days (*n* = 30), respectively

The mean tracking duration was 267 days (*SD* = 113 days, range: 26–687 days, median = 251 days, *n* = 78). The mean number of Argos transmissions received was 48,397 (range: 9,300–94,737 Argos transmissions, median = 47,975 Argos transmissions, *SD* = 17,298). The median tracking duration for the larger model of tags (291 days) was significantly longer than for the smaller model (237 days), as indicated by a Wilcoxon test (*Z* = 2.32, *p* < .05, *n* = 48 and 30, respectively) (Figure 2) (see Table S1). As of 19 November 2020, 7 of the 78 tags were still transmitting, with these ongoing deployments lasting from 340 to 356 days.

### 3.2 Battery exhaustion

**FIGURE 3:**
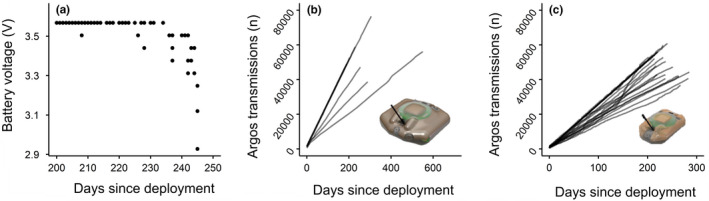
(a) An example of the drop in battery voltage indicative of battery exhaustion (final 50 days of deployment). In those cases where we did receive this voltage drop data, for (b) the larger model and (c) the smaller model of tag, the number of transmissions made by each tag versus the day of deployment. Battery exhaustion for the large and small tags occurred after a median of 352 days (range = 208–687 days) and 243 days (range: 210–275 days), respectively. Note there are fewer lines on panel (b) because the larger tags were attached to nesting turtles, which all migrated away from Diego Garcia and so well beyond line of sight of the Mote. Hence, far less data were received from these tags (data were relayed only via Argos once these turtles departed Diego Garcia), and hence, we were less likely to receive the voltage drop data compared with the smaller tags

For 21 of 71 tags that stopped transmitting, we received information on a drop in voltage indicative of battery exhaustion (Figure 3). We were more likely to receive this battery voltage drop data for those tags that remained within line of sight of the Mote, since the Mote increased data acquisition by 3–4 times compared with Argos‐only relay. For example, for 14 of the 20 immature turtles that remained within the lagoon at Diego Garcia, we received data in the final days of tracking showing a sharp drop in battery voltage, indicative of battery exhaustion. In contrast we received data on the battery voltage drops from only 7 of 51 turtles that migrated away from Diego Garcia when data were only relayed via Argos satellites.

Battery exhaustion for the large and small tags occurred after a mean of 53,635 Argos transmissions (range: 47,160–94,737 transmissions, *n* = 7) and 50,267 Argos transmissions (range: 40,542–60,810 transmissions, *n* = 14), respectively. Using the lower range value for the number of transmissions possible for each model of tag, for 20 of the remaining 34 large tags and 10 of the remaining 16 small tags that stopped transmitting, we concluded the batteries had exhausted. As of 19 November 2020, the seven ongoing deployments had passed the lower range value for the number of transmissions prior to battery exhaustion. When data from these tags stop being received, these tags will therefore most likely be included in the “battery exhaustion” category. So, we include them in that category here.

So, from our 2‐step procedure for assessing battery exhaustion (battery voltage and number of transmissions) we calculated that overall for 51 of 78 tags the batteries became exhausted (Table 1). There was a significant difference in the proportion of tags attached to nesting turtles versus immature turtles where battery failure was inferred as the reason why tags stopped relaying data (proportions 0.56 and 0.87, respectively, *G*_1_ = 0.89, *p* = <.01). Two equipped immature turtles where battery exhaustion was inferred were recaptured >1 year after the last transmission and inspection of the tag confirmed the batteries were exhausted.

**TABLE 1:** For the larger and smaller model of satellite tags, the number of tags where we inferred battery exhaustion and the number with other causes for the cessations of transmissions

Tag model	Battery exhaustion (*n*)	Saltwater switch failure (biofouling)	Unknown (*n*)	Total (*n*)
Splash 10 (large)	27	0	21	48
Splash 10 (small)	24	0	6	30
Total	51	0	27	78

### 3.3 Biofouling of saltwater switches

**FIGURE 4:**
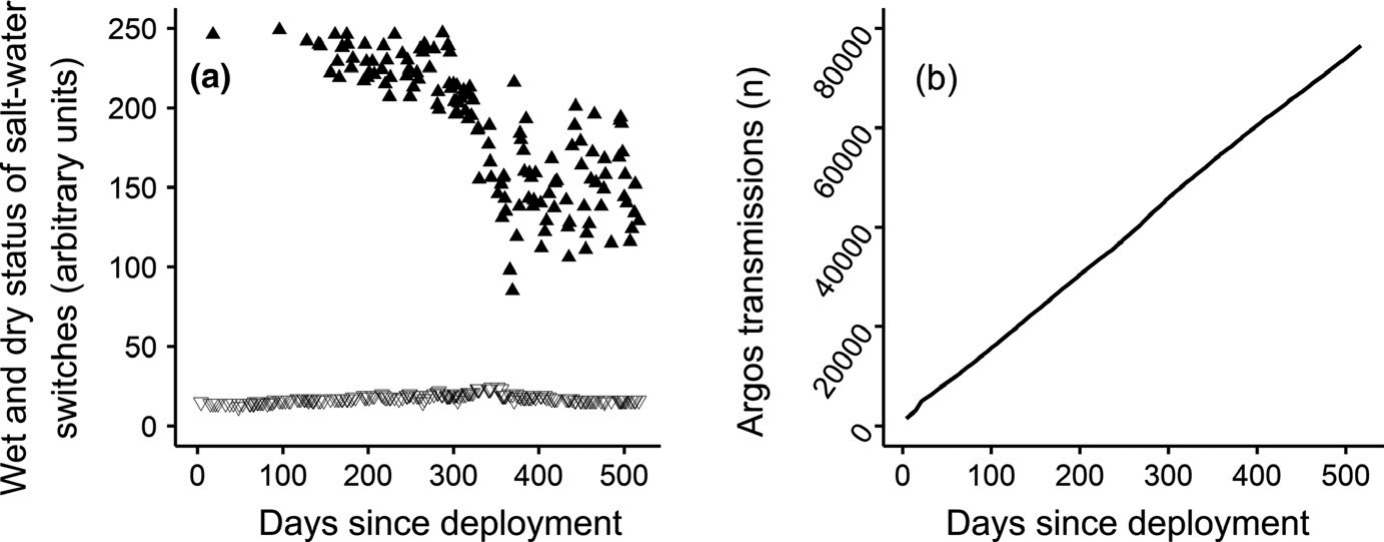
(a) For a single larger model of tag, an example of progressive biofouling of the saltwater switches, as indicated by the decline in the maximum “dry” state of the tag (closed triangles). Open triangles show the “wet” state, that is, the minimum dry state. However, there was always a clear difference between the “dry” and “wet” states, suggesting biofouling was not the reason for the cessation of transmissions. (b) For the same tag, the number of transmissions made by the tag plotted against the number of days since deployment. The number of transmissions made by the tag reached 76,552 after 517 days. This value is well above the lower range value for the number of transmissions before battery exhaustion (47,160 transmissions), suggesting that, in this example, battery exhaustion caused the tag to stop transmitting

For many tags (~30%), we received some evidence of biofouling of the saltwater switches, as indicated by a progressive decrease in their “dry” state (Figure 4). However, in all 23 cases, there was still a clear difference between the “dry” and “wet” states of the tag, and in all cases where biofouling was indicated, the reason for the cessation of transmission appeared to be battery exhaustion.

### 3.4 Other reasons for the end of tracking

**FIGURE 5:**
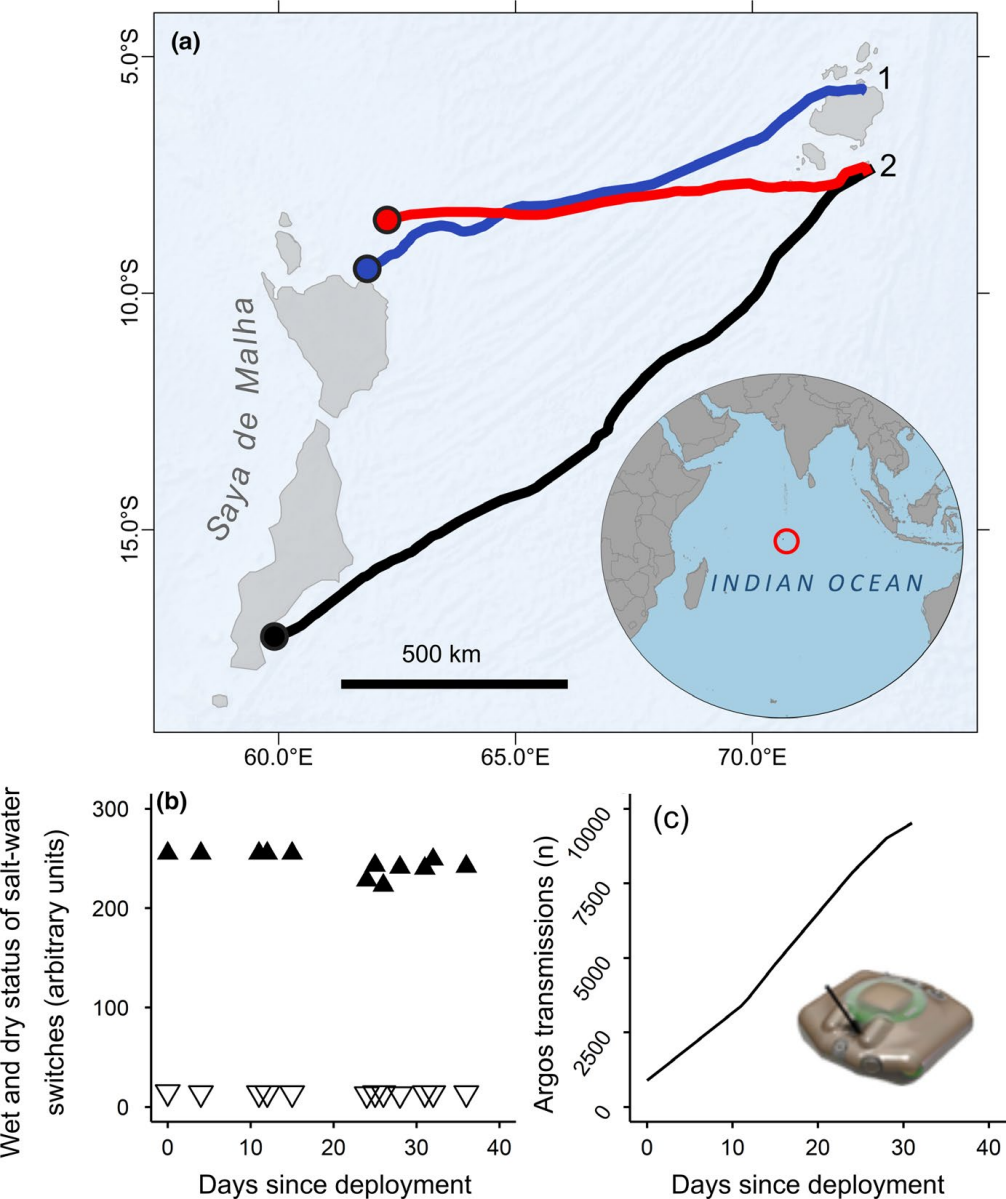
(a) Fastloc‐GPS tracks of 3 adult green turtles tagged and released from nesting beaches on (1) Nelson's Island and (2) Diego Garcia atoll in the Chagos Archipelago (position in the Indian Ocean shown by the red circle on the inset). Shaded grey areas represent submerged bank features <200 m depth. These turtles migrated westwards more than 1,200 km in the open ocean before tags stopped transmitting on encountering shallow waters associated with the Saya de Malha bank. In each case, transmissions ended before the lower range value for the number of transmissions expected for battery exhaustion. There was no indication in the diagnostic data from any of the tags of biofouling of the saltwater switches or battery exhaustion. Example plots for one tag show (b) the “dry” and “wet” states of the saltwater switches indicated by the closed and open triangles, respectively, and (c) the number of Argos transmissions the tag had made

In a number of cases (27 of 78), data from tags stopped being received before expected battery exhaustion. One tag, attached to an adult hawksbill turtle, appeared to come to the surface 18 days after deployment, as indicated by a sudden increase in the daily number of Argos messages received. At this time, the turtle was just off (within 5 km) the south coast of Diego Garcia. The tag then moved westwards at around 10 km per day for 8 days, before Argos signals stopped being received. Only 12,564 transmissions were made by this tag, well short of the expected battery duration. Data from three tags attached to adult green turtles stopped being received toward the end of long migrations. These three turtles traveled westwards from the Chagos Archipelago before tracking stopped as they all approached the Saya de Malha Bank, about 1,200 km from the nesting beaches where they were tagged (Figure 5). The number of transmissions from each tag was 20,486, 20,015, and 9,519, respectively, less than the lower range value for battery exhaustion (47,160 transmissions).

## 4 DISCUSSION

It is encouraging that many satellite tracking studies nowadays record fairly long tracking durations compared with earlier work. For example, Luschi et al. (1998) recorded tracking durations for six green turtles from Ascension Island of only 19–47 days and Hays and et al. (1991) tracked a loggerhead turtle for only 58 days. Likewise, early satellite tracking studies tracked an elk for 28 days (Craighead et al., 1972) and a basking shark for 17 days (Priede, 1984). Set against this backdrop, our median tracking duration of 251 days and some animals tracked for >500 days reflect many other recent sea turtle studies where tracking durations of >1 year have routinely been recorded, with sometimes tracking durations exceeding 1,000 days (Hays & Hawkes, 2018; Mingozzi et al., 2016). Similarly, compared with earlier studies, longer track durations are now routinely achieved for other taxa, such as sharks (Hammerschlag et al., 2011; Lea et al., 2015). There are probably many reasons for the general trend for longer tracking durations. Tag manufacturers and tag users have maintained a close dialogue to improve the robustness of tags. For example, the models of SPLASH tags that we used include a superflexible antenna with the base of the aerial sitting in small, well‐protected baffles to help reduce the probability of the aerial shearing off when scraped against rocks (Esteban et al., 2017; Hays & Hawkes, 2018). Attachment techniques have also improved. For example, early studies with sea turtles often used fiberglass cloth to secure the tag to the carapace of a turtle (e.g., Luschi et al., 1998), but nowadays much stronger epoxies tend to be used (Hays & Hawkes, 2018).

We used Argos tags that also allowed Fastloc‐GPS locations to be determined. The reason we used these tags is that Fastloc‐GPS provides locations generally accurate within a few 10s of meters of the true location, compared with Argos locations that are typically several km from the true location (Dujon et al., 2014; Thomson et al., 2017). Hence, we used the tracking data to assess the specific details of turtle movements (e.g., Hays et al., 2020). Nevertheless, the general approach we used applies equally to all types of Argos satellite tags that are widely deployed across many taxa (e.g., Hussey et al., 2015). Diagnostic data provided strong evidence that battery exhaustion was the main reason why tags stopped relaying data in our study, echoing findings reported by some others (e.g., Hanson et al., 2013; Hofman et al., 2019). In contrast, Hart et al. (2021) concluded that damage to their satellite tags was the main reason why data relay stopped. Based on the drop in tag battery voltage, Hart et al. (2021) concluded that only 4.1% of their satellite tags attached to turtles stopped relaying data due to battery failure. However, since the batteries fail quickly, often direct information on the drop in battery voltage is not received via Argos, as we showed here, with the detection of the battery voltage drop being more frequent when more battery status diagnostic information was received. Hence, we recommend a 2‐step procedure for assessing battery exhaustion using both battery voltage and number of transmissions, so that the prevalence of battery exhaustion is not underestimated.

Battery management is a trade‐off between acquiring more locations per day for shorter time periods, versus acquiring fewer daily locations but for longer. In our case, the interval between attempted GPS snapshots and the daily number of Argos transmissions were set at high values because a primary goal was to record highly detailed tracks so that the navigational performance of turtles could be assessed (see Hays et al., 2020 for outcomes). Clearly, if study objectives were more focused on the general long‐term patterns of movement, the battery management of tags could be adjusted to extend deployments (Patterson & Hartmann, 2011). So, our work serves to illustrate how objective analysis of why tags stop relaying data can be used to refine future tag deployments.

Our finding that battery exhaustion was the primary reason why tags stopped relaying data is noteworthy, because we can therefore conclude that tags often (a) remained attached to animals and (b) remained intact, over the duration of deployments. This finding is important because both tag detachment and tag damage have previously been implicated as important reasons for tag failure with sea turtles and other taxa (Hays et al., 2007; Hofman et al., 2019). Hence, our findings provide confidence concerning our tag design and attachment methods and suggest that with refinements to battery management, longer deployments will be possible. Similarly, diagnostic data revealed that although there was some biofouling of tags, this was not the reason why data relay stopped. Biofouling of tags has been implicated as an Achilles heel of many satellite tracking studies, particularly in warmer waters where epibiont settlement and growth may be quicker (Hays & Hawkes, 2018), with this issue being much less acute in colder areas (Henderson et al., 2020). Painting tags with antifouling paint will certainly help to alleviate the issue of biofouling and is now widely advocated (Hays & Hawkes, 2018). Examination and reporting the performance of saltwater switches across studies may help identify the best practice with regard to types of antifouling measures (e.g., the type of paint to use and application procedure).

It might be expected that tags attached to immature turtles might be more likely to detach due to the growth of the turtle and consequent expansion of the scutes that make up the carapace. For this reason, in some previous studies with immature turtles, a sheet of flexible neoprene has been placed under the tag as part of the attachment (e.g., Seney et al., 2010). However, in our case, battery failure rather than tag detachment was the most important reason for why tags stopped relaying data, particularly for immature turtles. High retention of the tags on immature turtles in our study might be attributed to the relatively short (generally <1 year) duration of tracking before battery failure, as well as the slow growth rates of immature turtles on Diego Garcia (Jeanne Mortimer, unpublished data). As might be expected, the larger model of tag containing more batteries generally relayed data for longer. For long deployments, the size of tags is often a balance between maximizing battery size versus minimizing the impact on tagged animals (Wilson & McMahon, 2006), and in common with most other studies, we opted for smaller tags on smaller turtles. Very long satellite tag deployments on small turtles may need particularly careful battery management, with some studies opting for severe duty cycling with tags only operating for <15% of the time (Christiansen et al., 2016). Particularly for such long deployments, it is very important to minimize the size of the attachment so that any negative impacts on the animal are reduced (Jones et al., 2013).

Satellite tagging is increasingly used to assess when animal mortality occurs across a range of species and habitats (Byrne et al., 2017; Klaassen et al., 2014; Loonstra et al., 2019; Murgatroyd et al., 2019). Using long‐term capture–mark–recapture protocols, annual mortality rates for sea turtles are generally reported to be around 0.25 (Schofield et al., 2020), that is, there is a 25% chance of a turtle alive at time “t” being dead at time “t+1year.” So, mortality of individuals might be expected to be a reason why tags sometimes stop relaying data. For example, assuming an annual mortality of 0.25, if 15 years of tracking data are accumulated (i.e., the sum of all individual tracking durations) then we would expect 0.25 × 15 = 3.75 mortality events to occur. Turtles may die for a number of reasons. They may suffer predation (e.g., Heithaus et al., 2007), may be killed in fisheries (Fossette et al., 2014) or by pollution such as plastics (e.g., Siegwalt et al., 2020). Unusual clusters of possible mortality might be cause for concern. In this regard, three green turtle tracks ended as individuals approached the Saya de Malha bank. All these tags appeared to be working perfectly when data relay stopped. It might be that all turtles suffered natural mortality, for example, through predation by tiger sharks, which are one of the few natural predators for adult green turtles (Heithaus et al., 2007). Alternatively, it might simply be that all three of these tags attached to green turtles failed because they detached or were damaged. However, this scenario seems unlikely given that all three green turtles were far from shallow water, which is generally where turtles are able to damage or remove transmitters through impacts with rocks. An alternative possibility is that these turtles were the victims of fishing bycatch. Closer examination of bycatch rates, for example, through onboard observers, might reveal if turtle bycatch is significant in that area.

In conclusion, we have shown here the value of assessing why tracking data from satellite tags stop being received. We advocate for these types of analysis regardless of the taxa being tracked, so that informed decisions can be made on how tag design and deployment can be refined to optimize data recovery. If researchers fully report the performance of their tracking methodologies and assess which are the most successful, this will help the field to move forward with the best practices for tag setup and attachments.

## CONFLICT OF INTEREST

None declared.

## AUTHOR CONTRIBUTIONS

**Graeme Hays:** Conceptualization (lead); data curation (supporting); formal analysis (equal); funding acquisition (equal); investigation (lead); methodology (equal); project administration (equal); writing–original draft (lead); writing–review and editing (lead). **Jacques‐Olivier Laloë:** Formal analysis (equal); writing–original draft (supporting); writing–review and editing (supporting). **Alex Rattray:** Data curation (lead); formal analysis (equal); visualization (lead); writing–original draft (supporting); writing–review and editing (supporting). **Nicole Esteban:** Funding acquisition (equal); investigation (lead); methodology (equal); project administration (equal); writing‐original draft (supporting); writing–review and editing (supporting).

## Supporting information

Table S1

## Data Availability

The data used in this study are published as Supplementary Information.
