# Supplementary material for: Why do Argos satellite tags stop relaying data?
Source: Ecol Evol. 2021 May 1;11(11):7093–101. doi: 10.1002/ece3.7558 (PMC8207149; doi:10.1002/ece3.7558)
Supplement: Supplementary file 1 — Table S1 [file ECE3-11-7093-s001.docx]

Supplementary Table 1. Hays et al. 2021 Why do Argos satellite tags stop relaying data? Ecology and Evolution.

For each tag, the model of transmitters (small versus large), the species and developmental stage of the turtle, tracking duration and number of Argos transmissions. Tags still working as of 17/11/2020 are indicated by the asterisk. CM = *Chelonia mydas* and EI = *Eretmochelys imbricata.*

| Argos ID | Species | Stage | Tag model | Release date | Tracking Duration (days) | Transmissions  (n) |
| --- | --- | --- | --- | --- | --- | --- |
| 21923 | CM | Adult | WC Splash10 (large) | 20/10/2012 | 227 | 34915 |
| 117568 | CM | Adult | WC Splash10 (large) | 23/10/2012 | 554 | 56139 |
| 117569 | CM | Adult | WC Splash10 (large) | 24/10/2012 | 452 | 45783 |
| 117570 | CM | Adult | WC Splash10 (large) | 24/10/2012 | 273 | 32729 |
| 117571 | CM | Adult | WC Splash10 (large) | 4/07/2015 | 223 | 27358 |
| 117570b | CM | Adult | WC Splash10 (large) | 6/07/2015 | 293 | 38381 |
| 117572 | CM | Adult | WC Splash10 (large) | 6/07/2015 | 252 | 42773 |
| 117573 | CM | Adult | WC Splash10 (large) | 7/07/2015 | 296 | 43467 |
| 21915 | CM | Adult | WC Splash10 (large) | 8/07/2015 | 352 | 67932 |
| 21923b | CM | Adult | WC Splash10 (large) | 9/07/2015 | 174 | 20479 |
| 117568b | CM | Adult | WC Splash10 (large) | 10/07/2015 | 382 | 69925 |
| 21914 | CM | Adult | WC Splash10 (large) | 11/07/2015 | 328 | 42227 |
| 117569b | CM | Adult | WC Splash10 (large) | 14/07/2015 | 89 | 20015 |
| 29358 | CM | Adult | WC Splash10 (large) | 15/07/2015 | 246 | 34019 |
| 41090 | CM | Adult | WC Splash10 (large) | 27/09/2017 | 92 | 20486 |
| 41089 | CM | Adult | WC Splash10 (large) | 28/09/2017 | 204 | 48011 |
| 41096 | CM | Adult | WC Splash10 (large) | 01/10/2017 | 199 | 49620 |
| 41098 | CM | Adult | WC Splash10 (large) | 01/10/2017 | 208 | 52655 |
| 41091 | CM | Adult | WC Splash10 (large) | 02/10/2017 | 193 | 48783 |
| 52227 | EI | Immature | WC Splash10 (small) | 29/06/2018 | 146 | 19593 |
| 52231 | EI | Immature | WC Splash10 (small) | 29/06/2018 | 176 | 44120 |
| 52224 | CM | Immature | WC Splash10 (small) | 30/06/2018 | 243 | 42879 |
| 52226 | EI | Immature | WC Splash10 (small) | 30/06/2018 | 266 | 45713 |
| 52232 | EI | Immature | WC Splash10 (small) | 30/06/2018 | 265 | 49551 |
| 41086 | CM | Adult | WC Splash10 (large) | 02/07/2018 | 206 | 51258 |
| 41097 | CM | Adult | WC Splash10 (large) | 03/07/2018 | 687 | 74904 |
| 41100 | CM | Adult | WC Splash10 (large) | 04/07/2018 | 289 | 64956 |
| 41102 | CM | Adult | WC Splash10 (large) | 04/07/2018 | 195 | 49047 |
| 41082 | CM | Adult | WC Splash10 (large) | 05/07/2018 | 182 | 43965 |
| 52214 | CM | Adult | WC Splash10 (large) | 06/07/2018 | 301 | 74450 |
| 52215 | CM | Adult | WC Splash10 (large) | 06/07/2018 | 169 | 42831 |
| 52202 | CM | Adult | WC Splash10 (large) | 07/07/2018 | 326 | 81177 |
| 52189 | CM | Adult | WC Splash10 (large) | 08/07/2018 | 230 | 56308 |
| 52221 | CM | Adult | WC Splash10 (large) | 08/07/2018 | 308 | 73910 |
| 52222 | CM | Adult | WC Splash10 (large) | 09/07/2018 | 235 | 58832 |
| 52223 | CM | Adult | WC Splash10 (large) | 09/07/2018 | 385 | 94737 |
| 52234 | EI | Immature | WC Splash10 (small) | 13/07/2018 | 275 | 41667 |
| 52237 | EI | Immature | WC Splash10 (small) | 13/07/2018 | 267 | 52035 |
| 52252 | EI | Immature | WC Splash10 (small) | 13/07/2018 | 237 | 59586 |

Supplementary Table 1. (continued)

| Argos ID | Species | Stage | Tag model | Release date | Tracking Duration (days) | Transmissions |
| --- | --- | --- | --- | --- | --- | --- |
| 52253 | CM | Immature | WC Splash10 (small) | 14/07/2018 | 210 | 52870 |
| 52236 | EI | Immature | WC Splash10 (small) | 15/07/2018 | 242 | 60810 |
| 64789 | EI | Immature | WC Splash10 (small) | 23/11/2018 | 219 | 47938 |
| 64790 | EI | Immature | WC Splash10 (small) | 23/11/2018 | 221 | 55877 |
| 64791 | EI | Immature | WC Splash10 (small) | 23/11/2018 | 221 | 55754 |
| 64792 | EI | Immature | WC Splash10 (small) | 24/11/2018 | 103 | 23293 |
| 64793 | EI | Immature | WC Splash10 (small) | 24/11/2018 | 213 | 53751 |
| 64794 | EI | Immature | WC Splash10 (small) | 25/11/2018 | 233 | 52613 |
| 64795 | EI | Immature | WC Splash10 (small) | 26/11/2018 | 215 | 54025 |
| 64797 | EI | Immature | WC Splash10 (small) | 26/11/2018 | 253 | 47170 |
| 64798 | EI | Immature | WC Splash10 (small) | 26/11/2018 | 268 | 36738 |
| 64802 | EI | Immature | WC Splash10 (small) | 26/11/2018 | 239 | 50381 |
| 52218 | EI | Adult | WC Splash10 (large) | 28/11/2018 | 474 | 56925 |
| 52217 | EI | Adult | WC Splash10 (large) | 29/11/2018 | 259 | 47160 |
| 52220 | EI | Adult | WC Splash10 (large) | 30/11/2018 | 457 | 73284 |
| 64783 | EI | Adult | WC Splash10 (large) | 01/12/2018 | 514 | 70856 |
| 64786 | EI | Adult | WC Splash10 (large) | 01/12/2018 | 525 | 80036 |
| 178882 | EI | Immature | WC Splash10 (small) | 19/03/2019 | 237 | 40542 |
| 178881 | EI | Immature | WC Splash10 (small) | 19/03/2019 | 266 | 42137 |
| 178884 | EI | Immature | WC Splash10 (small) | 19/03/2019 | 236 | 42780 |
| 182221 | CM | Adult | WC Splash10 (large) | 07/07/2019 | 39 | 9519 |
| 182218* | EI | Adult | WC Splash10 (large) | 29/11/2019 | 356 | 73472 |
| 182219* | EI | Adult | WC Splash10 (large) | 30/11/2019 | 355 | 61661 |
| 182220* | EI | Adult | WC Splash10 (large | 30/11/2019 | 355 | 84943 |
| 182222 | EI | Adult | WC Splash10 (large) | 30/11/2019 | 26 | 12564 |
| 64784 | EI | Adult | WC Splash10 (large) | 30/11/2019 | 249 | 39749 |
| 64788 | EI | Adult | WC Splash10 (large) | 02/12/2019 | 218 | 46582 |
| 64787 | EI | Adult | WC Splash10 (large) | 03/12/2019 | 293 | 38690 |
| 182213* | EI | Adult | WC Splash10 (large) | 03/12/2019 | 352 | 42514 |
| 182217* | EI | Adult | WC Splash10 (large) | 04/12/2019 | 351 | 60894 |
| 178883 | EI | Adult | WC Splash10 (small) | 04/12/2019 | 76 | 14375 |
| 182212 | EI | Adult | WC Splash10 (small) | 05/12/2019 | 219 | 52079 |
| 182208 | EI | Adult | WC Splash10 (small) | 05/12/2019 | 310 | 48076 |
| 178885 | EI | Adult | WC Splash10 (small) | 07/12/2019 | 289 | 40621 |
| 182211 | EI | Adult | WC Splash10 (small) | 12/12/2019 | 37 | 9300 |
| 182210 | EI | Adult | WC Splash10 (small) | 12/12/2019 | 302 | 39100 |
| 182209 | EI | Adult | WC Splash10 (small) | 13/12/2019 | 286 | 44288 |
| 182214* | EI | Adult | WC Splash10 (large) | 14/12/2019 | 341 | 44556 |
| 182215* | EI | Adult | WC Splash10 (large) | 15/12/2019 | 340 | 49838 |
